# Investigation of quantitative structural changes in astrocyte cells after laser-induced shockwave

**DOI:** 10.1117/1.BIOS.2.4.045002

**Published:** 2025-12-03

**Authors:** Pegah Pouladian, Janelle Ho, Nicolas Perez, Nicole M. Wakida, Veronica Gomez-Godinez, Daryl Preece

**Affiliations:** a University of California Irvine, Beckman Laser Institute, Department of Biomedical Engineering, Irvine, California, United States; b University of California San Diego, Institute of Engineering in Medicine, San Diego, California, United States

**Keywords:** quantitative phase microscopy, astrocyte morphology, laser-induced shockwave, traumatic brain injury, cellular response to mechanical stress

## Abstract

**Significance:**

Traumatic brain injury (TBI) arises from external forces impacting the brain, leading to outcomes that range from mild to severe. Despite continuous scientific advancements, TBI remains a significant cause of physical impairment and mortality. In recent years, blast-induced TBI (bTBI) has become more prominent due to the use of explosive devices, necessitating accurate models to simulate and study the effects of shockwaves on brain tissue. To better understand bTBI at the cellular level, various models have been proposed. Laser-induced shockwaves (LISs) have emerged as an effective method to simulate bTBI in a controlled environment by generating shockwaves through pulsed laser-induced plasma formation.

**Aim:**

We introduce a cost-efficient method to investigate cellular morphology changes in response to mechanical stress by combining LIS and quantitative phase microscopy (QPM).

**Approach:**

QPM, a label-free imaging technique, facilitates quantitative visualization of cellular dynamics. The integration of LIS and QPM enabled the precise assessment of type 1 astrocyte cells under shear stress, revealing both immediate and sustained morphological changes.

**Results:**

Key findings include significant alterations in some morphological features such as surface area to volume ratio (p<0.001) immediately post-LIS, which returned to baseline within 2 h, and lasting changes in features such as circularity (p<0.001), suggesting prolonged cellular adaptation. These insights provide a deeper understanding of how mechanical stimuli affect astrocyte morphology, offering potential pathways for targeted therapeutic strategies in TBI and related neurological disorders.

**Conclusions:**

The integrated QPM-LIS approach serves as a powerful tool for studying quantitative cellular dynamics, opening the door to further investigations of astrocyte and other brain cell morphologies in response to mechanical forces, with broad implications for neurological research and therapy development.

Statement of Discovery This work shows that astrocyte cells undergo previously unobserved tiny morphological changes seconds after damage, which can be observed with quantitative phase microscopy and image processing algorithms. These changes can be used to give immediate indications of cell health and have implications for drug testing and biomarker discovery.

## 1 Introduction

Traumatic brain injury (TBI) occurs when an external force disrupts brain function, leading to outcomes that can range from full neurological recovery to severe impairment or death. This injury is particularly prevalent among young people and continues to be a leading cause of disability and mortality.^1^ In recent years, blast-induced TBI (bTBI) has become more prominent due to the use of explosive devices, necessitating accurate models to simulate and study the effects of shockwaves on brain tissue.^2^^,^^3^ Several models have been developed to replicate the effects of bTBI, with laser-induced shockwaves (LISs) emerging as a promising method to simulate the mechanical forces of blast injuries. LIS generates cavitation bubbles through pulsed laser interaction with fluid, creating shockwaves that apply controlled mechanical stress to cells,^4^ allowing for detailed investigation of cellular damage.

Previous studies highlight LIS as a valuable TBI simulation model. Selfridge et al.^5^ showed that LIS can replicate TBI forces at the cellular level, enabling the study of neuronal death and repair mechanisms. Gomez Godinez et al.^6^ demonstrated LIS’s utility with genetically encoded biosensors to monitor molecular responses such as calcium ion (Ca^2+^) dynamics, offering insights into how mechanical forces influence cellular signaling in TBI. Similarly, Carmona et al. developed an optical system to visualize and measure shockwave dynamics at the single-cell level, examining early cellular responses to shear stress.^7^ These findings confirm LIS as a robust model for studying TBI *in vitro*. Building on this foundation, our study specifically explores astrocyte responses to mechanical stress, with a focus on changes in cell morphology. Astrocytes, the most abundant glial cells in the central nervous system, are essential for maintaining homeostasis, regulating ion and water balance, and supporting neuronal function.^8^ These cells are also involved in responding to brain injuries through a process called astrogliosis, which involves changes in cell morphology and function in response to mechanical stress. Astrocyte morphology is highly complex, with star-shaped cells extending multiple branching processes that interact with neurons and form gliapil regions, where synaptic activity occurs.^9^^,^^10^ Disruptions in astrocyte structure and function have been implicated in several neurodegenerative diseases, including Alzheimer’s and Parkinson’s diseases, where astroglial hypertrophy and other morphological changes have been observed.^11^^,^^12^

The morphological response of astrocytes to mechanical stress, particularly in the context of TBI, remains an area of active research. Understanding astrocyte morphology at the single-cell level is essential for linking cellular-scale mechanical injury to broader neuropathological outcomes. Morphological changes in astrocytes are not merely structural but reflect shifts in astrocyte function, including pro-inflammatory signaling, neurotransmitter uptake, and support for neuronal survival. These early morphological responses are among the first detectable signs of reactive astrogliosis and can influence the trajectory of injury resolution or secondary degeneration. Furthermore, single-cell analysis enables detection of heterogeneous responses that may be masked in bulk measurements, offering critical insight into how localized mechanical stress can lead to divergent cellular fates and influence long-term tissue remodeling in TBI. Several studies have established that morphological changes in astrocytes are among the earliest responses to traumatic injury and are predictive of downstream pathological processes. For example, astrocyte hypertrophy and process retraction are hallmarks of early reactive gliosis, which often precedes the formation of glial scars that can inhibit axonal regeneration and contribute to cognitive deficits.^13^ Furthermore, changes in astrocytic morphology are closely linked to shifts in inflammatory signaling, extracellular ion buffering, and blood–brain barrier integrity—factors known to influence long-term functional outcomes following TBI.^14^ Thus, quantifying these morphological dynamics provides not only a readout of cellular injury but also a potential biomarker for predicting injury progression and guiding therapeutic intervention. Quantitative phase microscopy (QPM) provides a powerful, label-free imaging method for observation of transparent cells, allowing detailed measurement of cellular morphology via phase shifts that reflect the optical path length (OPL = refractive index × thickness). Although QPM does not directly resolve the three-dimensional refractive index distribution, assuming a uniform average refractive index enables estimation of height and volumetric features, an approach commonly used in QPM studies.^15^ In this study, we use QPM to monitor and quantify the morphological changes in astrocytes following LIS exposure, providing a detailed analysis of their response to mechanical injury. Expanding on previous research that combined LIS and QPM to study cellular responses,^15^ this work focuses on the structural dynamics of astrocytes (AST1 cells) under TBI-like forces, with the goal of uncovering how mechanical injury affects cellular architecture and repair mechanisms. This approach offers a cost-effective, *in vitro* method to simulate TBI and capture detailed cellular changes, providing an accessible alternative to more complex and expensive models traditionally used in brain injury research.

Our study reveals significant morphological changes in astrocytes following exposure to LIS, including alterations in cell shape, height, and surface characteristics. These observations shed light on the dynamic processes driving astrogliosis and demonstrate how astrocytes adjust their structure in response to mechanical injury. By integrating LIS with QPM, this approach provides a novel and detailed method of studying cellular injury, offering valuable insight into the behavior of astrocytes and their critical role in central nervous system repair after traumatic brain injury.

## 2 Materials and Methods

### 2.1 Cell Preparation

An established astrocyte type I (Ast-1) line (clone CRL-2541) was received directly from ATCC. Ast-1 cells were cultured in advanced DMEM media supplemented with 2% FBS and 1% GlutaMAX. Cells were plated on 35-mm glass-bottom imaging plates to ensure optimal conditions for imaging and LIS application. Cell density was maintained consistently across experimental conditions, with $∼35\;k\;\text{cells}/{\mathrm{cm}}^{2}$, ensuring that potential mechanical coupling effects among neighboring cells did not unduly influence the results. Future experiments will explore the role of varying cell density to assess its impact on mechanical coupling during LIS exposure.

### 2.2 Quantitative Phase Microscopy and Laser-Induced Shockwave

Our system integrates a custom-built QPM with LIS capabilities to enable simultaneous, label-free imaging and mechanical stimulation of live cells. This configuration facilitates quantitative monitoring of cellular responses, morphological and biophysical, to precisely controlled laser-generated shockwaves.

The QPM operates on a Linnik interferometer design, using two identical 20×/0.4 NA objective lenses for sample and reference arms. Illumination is provided by a red LED source (λ=633  nm), and the sample beam power at the back aperture is limited to 0.32 mW to avoid phototoxic effects during extended imaging sessions.^16^ The LIS system utilizes a Q-switched diode-pumped solid-state laser (Flare NX, Coherent) emitting 1.5-ns pulses at 1030 nm with a maximum repetition rate of 2000 Hz. Laser energy per pulse was measured at 60  μJ using a calibrated thermal power sensor. The laser beam was expanded, power-controlled using a half-wave plate and polarizing beamsplitter, and focused through the objective onto the sample medium. The beam profile was characterized by taking a Z-stack of the Rayleigh range and beam waist characterized via Gaussian fitting, yielding a full-width at half-maximum of 3.1  μm. From this, the beam radius was calculated as 1.27  μm.

Using these parameters, the peak irradiance at the focal point was estimated at $1.02\times 10^{11}\;W/{\mathrm{cm}}^{2}$, surpassing the breakdown threshold for water and enabling consistent LIS generation. The plasma volume produced by the laser was ellipsoidal, ∼13  μm in length and 8  μm in diameter. The resulting cavitation bubble reached a maximum radius of ∼21  μm, corresponding to an estimated energy release of 3.78 nJ, estimated using the Rayleigh model.^17^

**Fig. 1:**
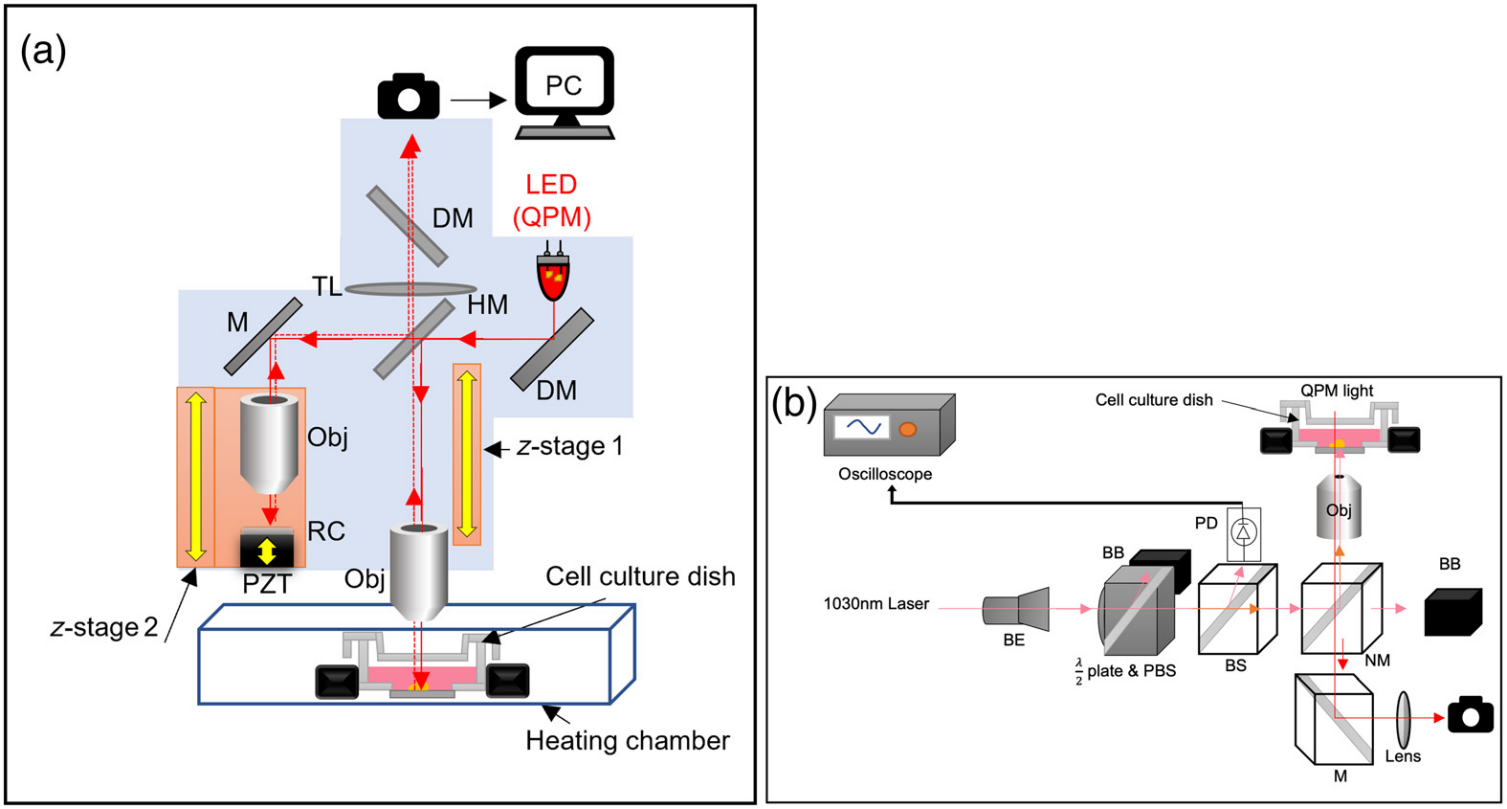
Schematic diagram of the integrated (a) QPM and (b) LIS setup. The system enables laser delivery from below while performing high-resolution interferometric imaging from above.

To maintain optical compatibility with the QPM system and avoid energy loss or thermal damage from the mirrors, the laser was introduced from below using standard glass-bottom dishes. Unlike earlier iterations that used thick dielectric mirrors, this setup minimized optical aberration and allowed tighter laser focusing with minimal working distance constraints (Fig. 1).

This integrated platform allows precise application of mechanical shear stress to astrocytes or other neural cells while capturing high-resolution phase shifts and dry mass changes, critical for modeling *in vitro* TBI and other mechanobiological processes. Additional technical and characterization details are provided in our earlier work.^15^

### 2.3 Cell Segmentation

Image segmentation, especially precise cell boundary delineation, is a major challenge in cell studies and is essential for accurate downstream analyses across scales.^18^ It involves dividing an image into regions corresponding to individual cells,^19^ enabling tasks such as cell counting,^20^ tracking,^21^ and morphology analysis.^22^ Effective segmentation requires accurate masks that clearly define borders and separate adjacent cells. Various methods have been proposed to achieve this, which will be discussed in Sec. 2.4. The interference cell images, captured by the QPM, were processed into final real height maps for each cell culture dish at various time points. The wrapped phase images were constructed from the fringe images by the algorithm developed by Toyohiko Yamauchi et al.^23^ The Goldstein method was used to unwrap the images into quantitative phase images.^24^^,^^25^ The phases were then converted to real height images.^15^ We note that these height maps represent estimations because QPM measures optical path length (refractive index × thickness). In this work, we assume a uniform average refractive index for the astrocyte cytoplasm, a standard approximation in QPI studies, to derive height and volumetric estimates. The images were then imported as sequences into ImageJ, and rectangles were manually drawn around the cells at each time point to define their bounding boxes. The “measure” tool within the “analyze” section was then used to obtain measurements of the position, height, and width of each rectangle. The resulting data were saved as CSV files and subsequently loaded into MATLAB for further processing, specifically to crop the real height map images.

**Fig. 2:**
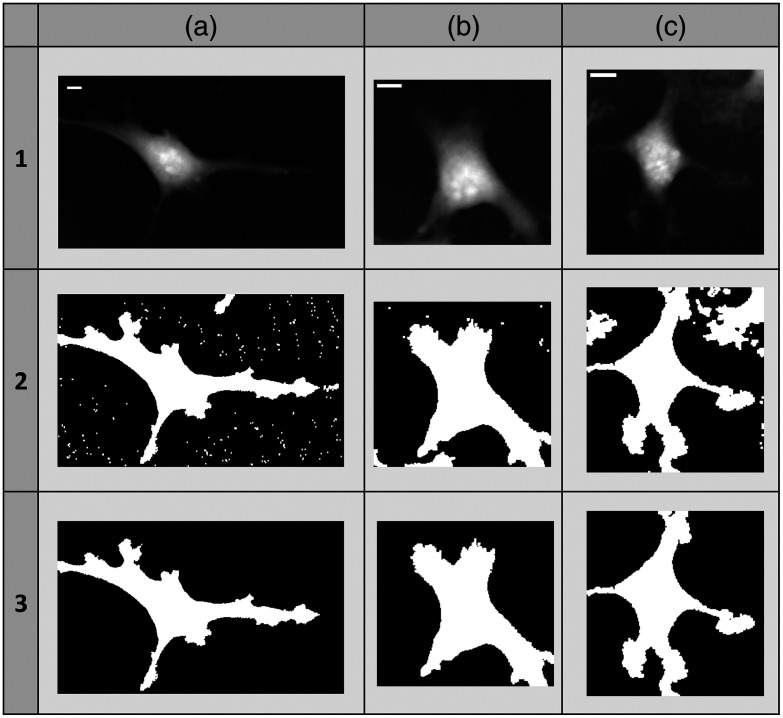
Three example cells (a, b, and c) and their respective masks. Each cell has three corresponding images: (1) grayscale image, (2) mask image created with ImageJ, and (3) final processed image generated with MATLAB.

The cropped images, each representing an individual cell at a specific time point, underwent batch processing to generate masks. The batch processing involved applying the “auto local threshold” on images using the “median” method with a 50-pixel radius. This was followed by one round of erosion to eliminate noise and small particles and one round of dilation. In instances where cells within the bounding box were closely packed, the process of isolating the desired cell may have resulted in the generation of more than one object in the mask. To address this, the masks were imported into MATLAB for additional image processing. The “regionprops” function was utilized to identify the object with the highest surface area, corresponding to the main cell in the image. The other objects were then eliminated, resulting in a mask that contained only the object corresponding to the desired cell. Figure 2 illustrates four cells alongside their respective images: a grayscale image, a mask image created with ImageJ, and a final processed image generated with MATLAB.

### 2.4 Verification of Automated Segmentation

To assess the accuracy of the automated segmentation, we compared feature values computed from automated and manually annotated masks on a randomly selected set of 20 astrocyte cells. Manual masks were generated by trained annotators from QPM images using boundary tracing.

We evaluated key morphological features, and the percent error was calculated as

$$\text{Percent error}=|\frac{F_{\text{auto}}-F_{\text{manual}}}{F_{\text{manual}}}|\times 100.$$

Table 1 summarizes the average error per feature across all manually compared masks.

**Table 1:** Average percent error between automated and manual segmentation across 20 cells.

Feature	Avg. error (%)
Volume	7.07
Surface area	0.40
Surface area to volume ratio	5.87
Eccentricity	13.19
Projected area to volume ratio	10.90

**Fig. 3:**
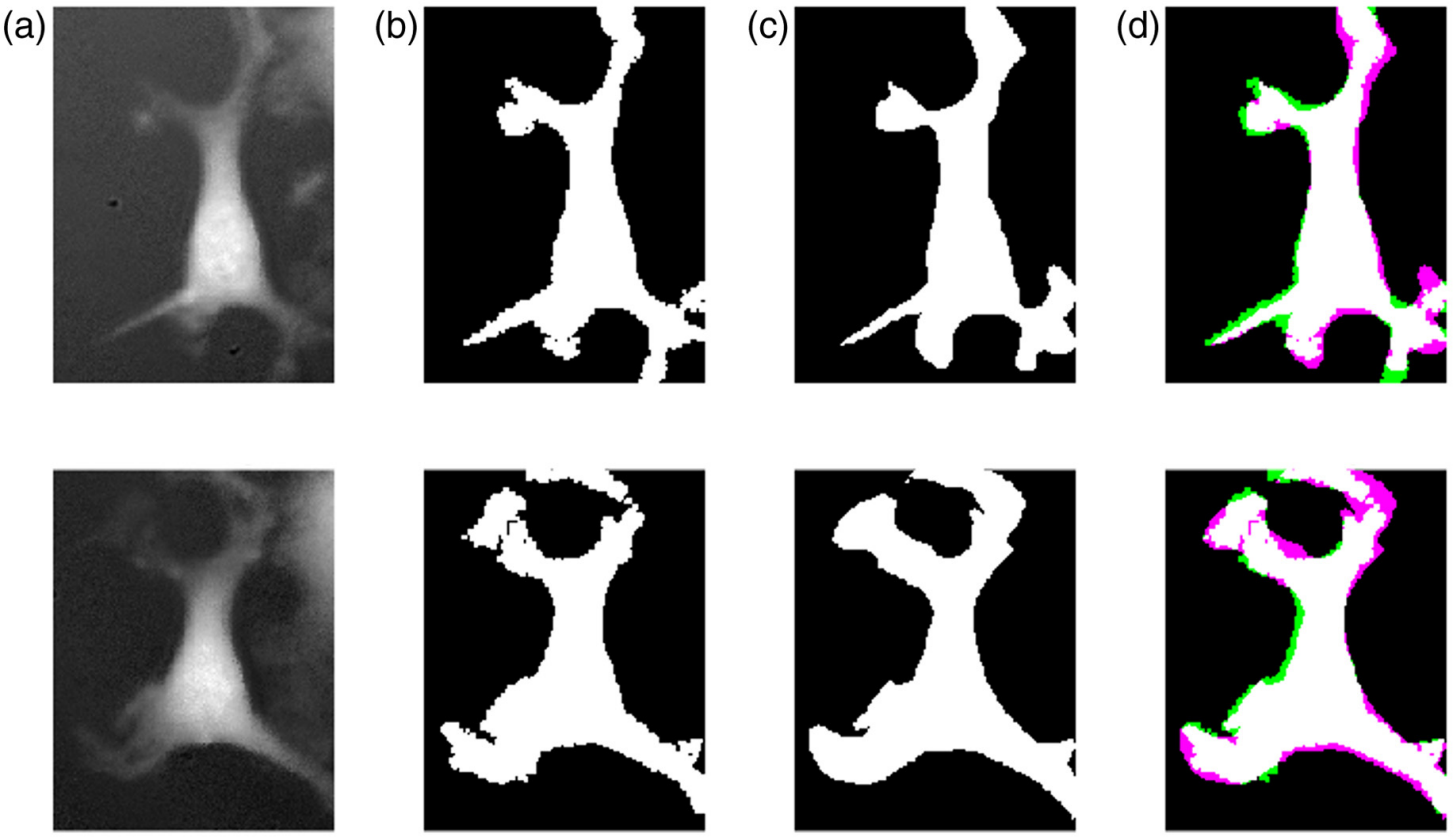
Comparison of automated and manual segmentation. (a) Cell image. (b) Automatic mask. (c) Manual mask (hand-traced). (d) Overlay illustrating boundary differences. Automated segmentation captures fine structural detail better than manual annotation.

Although geometric features such as eccentricity showed higher variation, biologically relevant metrics such as volume and dry mass were closely matched. Notably, manual segmentation of astrocytes is inherently challenging due to their irregular and diffused morphology. Figure 3 highlights this difference, showing that the automated pipeline delineates cellular boundaries more consistently and accurately.

### 2.5 Background Correction

To eliminate any background interference caused by uneven exposure, a mask generated through ImageJ processing was utilized as a guide for calculating the average background height. The mask included all particles within the bounding box, and the remaining part of the image represented the background. The calculated average was then subtracted from the entire image, resulting in a background-corrected image.

### 2.6 Feature Extraction

Numerous morphological features could be extracted from height map images. Features that were considered for this study were dry mass, surface area, surface area to volume ratio, surface area to dry mass ratio, height variance, height kurtosis, height skewness, eccentricity, circularity, perimeter, perimeter to projected area ratio, and complexity score. With the exception of the last four features, all measurements were derived from the parameters detailed in the study by Girshovitz and Shaked^26^ A comprehensive elucidation of the newly incorporated features is provided in the following.

• Circularity. Determined using the circularity feature in MATLAB’s “regionprops” function, circularity evaluates the similarity of the cell’s shape to that of a perfect circle. This measure ranges from 0 to 1, with 1 representing a perfect circle. Higher circularity values indicate a closer resemblance to a circular shape. Deviations from circularity can suggest alterations in cell structure.
• Perimeter. Perimeter (perim) is a feature that represents the total length of the outer boundary of a cell. It provides information about the cell’s extent and boundary complexity. Changes in the perimeter may indicate variations in cell size or irregularities in cell shape.
• Perimeter to area ratio. PerimDivArea is a feature calculated by dividing the perimeter of a cell by its area. This ratio offers insight into the cell’s boundary irregularity relative to its overall size. Higher values may suggest a more complex and convoluted cell boundary compared with its size.
• Complexity score. This score is calculated based on the convex hull algorithm. In addition, the convex hull represents the smallest convex polygon that encloses the given cell, providing insight into the cell’s overall spatial characteristics. It measures the difference in the projected area of the cell and its convex hull, divided by the convex hull shape area. The less spikey the cell, the lower this score would be
  $$\text{Complexity score}=\frac{\text{Convex hull area}-\text{Cell area}}{\text{Convex hull area}}.$$

**Fig. 4:**
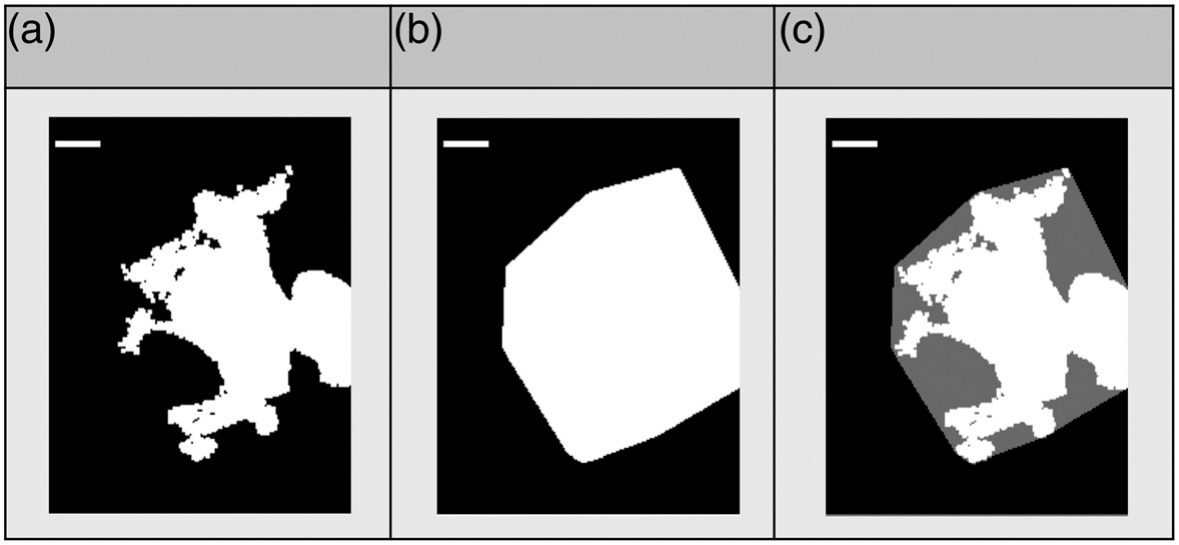
Example of the (a) binary mask of a cell, (b) its convex hull image, and the (c) superimposed image of the binary mask and the convex hull image. The scale bar is 10  μm.

Figure 4 displays an example of the binary mask of a cell (a), its convex hull image (b), and the superimposed image of the binary mask and the convex hull image (c).

**Fig. 5:**
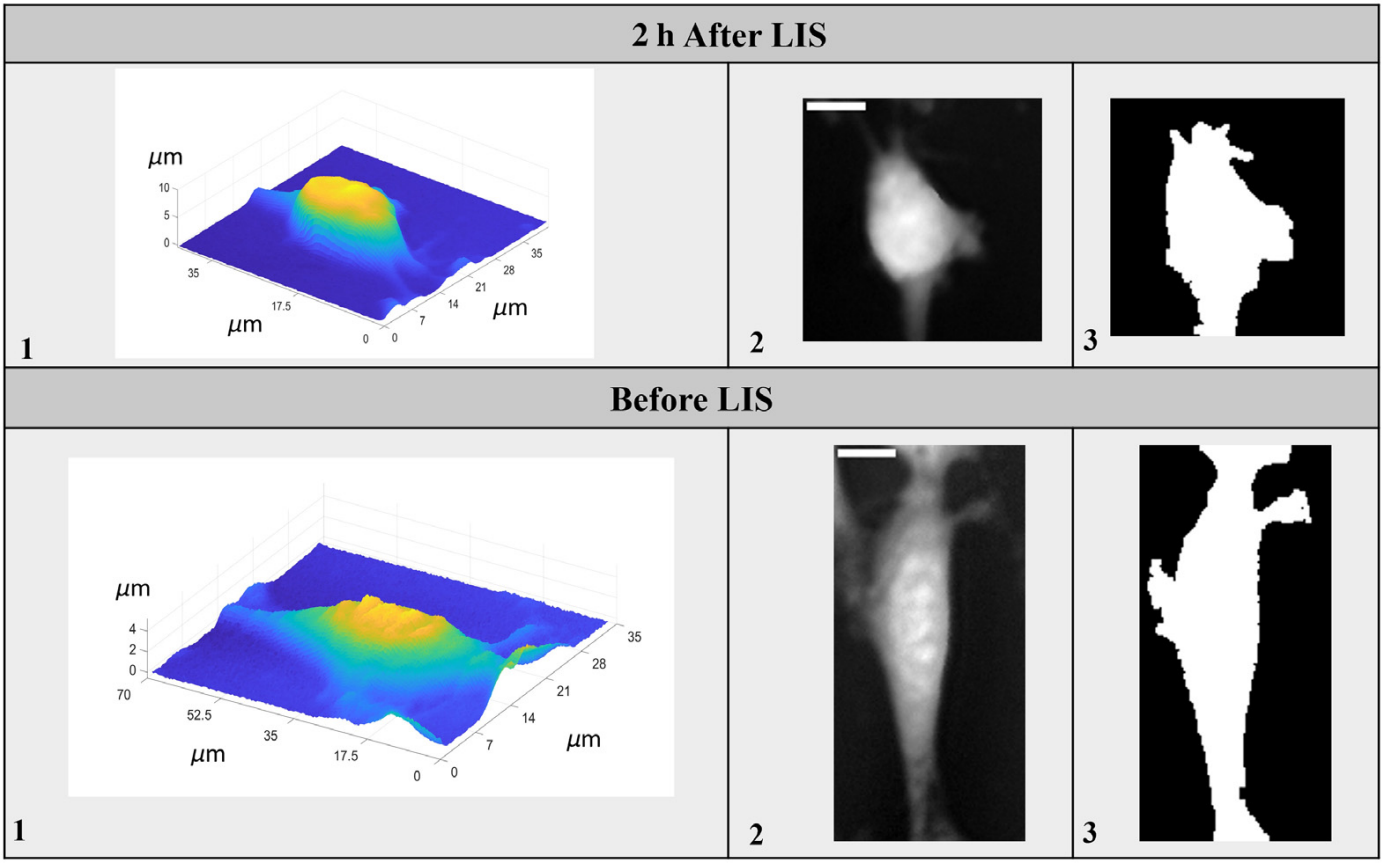
(1) Phase-derived topographical height maps of a cell, (2) grayscale image, and (3) corresponding final masks, 2 h after LIS and before LIS.

These features collectively contribute to our understanding of cell biology, enabling us to assess the effects of various conditions, treatments, or genetic manipulations on cellular structure and function. Figure 5 displays two phase-derived topographical height maps of a cell (1), the grayscale images of a cell (2), and the corresponding masks (3) at two different time points: (I) before LIS and (II) 2 h after LIS. Table 2 shows the table of corresponding feature values for each image.

**Table 2:** Comparison of features for the cell shown in Fig. 5, before and 2 h after LIS.

Parameter	2 h after LIS	Before LIS
Circularity	0.44	0.22
Volume	$2061.8\;\mu m^{3}$	$1779.6\;\mu m^{3}$
Projected area	$508.5\;\mu m^{2}$	$802.1\;\mu m^{2}$
Dry mass	11.5 p gr	9.9 p gr
Surface area	$14729.0\;\mu m^{2}$	$19845.7\;\mu m^{2}$
Surface area to volume ratio	7.1 1/μm	11.2 1/μm
Surface area to dry mass ratio	$1285.9\;\mu m^{2}/p\;\mathrm{gr}$	$2007.3\;\mu m^{2}/p\;\mathrm{gr}$
Projected area to volume ratio	0.25 1/μm	0.45 1/μm
Sphericity	$0.0008\;\mu m^{1/3}$	$0.0004\;\mu m^{1/3}$
Height variance	$9.9\;\mu m^{2}$	$1.8\;\mu m^{2}$
Height kurtosis	1.5	1.9
Height skewness	0.2	0.1
Eccentricity	0.26	0.45
Perimeter	361.4 μm	643.2 μm
Perimeter to area ratio	0.71 1/μm	0.80 1/μm
Complexity score	0.20	0.43

### 2.7 Control Group

AST1 cells were maintained and observed within a temperature-controlled chamber designed specifically for the QPM setup. The chamber’s environmental conditions, including air composition, were precisely regulated using an Ibidi gas mixer, which ensured a stable atmosphere throughout the experiment. Key parameters such as humidity (kept constant at 80%) and ${\mathrm{CO}}_{2}$ concentration (set at 5%) were tightly controlled to replicate the cells’ natural environment and minimize stress or alterations in their behavior.

During the observation period, the AST1 cells were subjected to a time-lapse imaging protocol. Images were captured every 20 min for a total duration of 2 h, with no interference or disruption to the cells’ natural activity. In total, 84 cells from 3 separate plates were analyzed and processed for segmentation and feature extraction.

We chose to use separate control cells rather than imaging the same cells before and after LIS treatment to maintain consistent imaging conditions and timelines for both the control and LIS groups. This decision ensured that both groups were exposed to identical environmental and imaging conditions throughout the experiment.

To achieve this, the entire experimental setup was designed so that both the LIS and control groups were placed under the microscope, and outside the incubator for the same total duration−∼2.5  h. Both groups were housed in the same custom-built imaging chamber, which preserved a stable environment and eliminated variability due to temperature, humidity, or ${\mathrm{CO}}_{2}$ fluctuations. This careful synchronization ensured that any observed cellular changes could be attributed solely to the LIS intervention, rather than to differences in handling or external conditions.

Longitudinal imaging of the control group further confirmed the stability of their morphology and biophysical features across the imaging period. No statistically significant changes were observed, validating their use as a baseline reference for comparison against the LIS-treated cells.

### 2.8 Laser-Induced Shockwave Group

The AST1 cells were kept under conditions similar to those of the control group, with the exception that they were subjected to a laser-induced shockwave. This experiment involved a continuous imaging process that started 20 min before the shockwave and lasted for 2 h afterward. The resulting quantitative phase images were analyzed to detect and measure various aspects of cell morphology. To evaluate the effect of the shockwave on the cells, we focused on their morphology 1 min before, 10 min after, and 2 h after the shockwave. By doing so, we could identify any rapid or gradual changes or responses induced by the shockwave. This approach allowed us to gain a deeper understanding of how cells react and adapt to sudden environmental changes under these unique experimental conditions. A total of 204 cells collected from 4 dishes were subjected to analysis.

### 2.9 Statistical Analysis

The statistical tests performed to assess the changes in AST1 cell parameters within the control and LIS group were performed through MATLAB using the Wilcoxon signed-rank test, a nonparametric test suitable for paired data. As the measurements were obtained from the same set of cells during the imaging period, the data are inherently dependent on each other. The Wilcoxon signed-rank test is well-suited for such paired comparisons, providing a robust analysis that does not assume a normal distribution of the data. This method accounts for the paired nature of the observations, which makes it appropriate for detecting subtle changes in the parameters of AST1 cells over time. The Mann–Kendall tau-b test^27^ was also performed for data trend analysis. This is a non-parametric statistical method employed for the detection of monotonic trends in time series data. It specifically assesses the presence of an upward or downward trend over time without assuming any underlying distribution of the data. The tau-b statistic represents the magnitude of the trend, and the p-value indicates the statistical significance of that trend.

## 3 Results

### 3.1 Control Group

For the control group, AST1 cells were subjected to 2 h of imaging. The average value for each feature for the total of 84 cells is summarized in Table 3. After 2 h of imaging, a comparison of the feature values was carried out, as presented in Table 4. The statistical analysis (all p>0.05) confirmed that no significant morphological changes occurred in the control group during the experimental period. This quantitative stability validates the control group as a reliable baseline for comparison with LIS-treated cells.

**Table 3:** AST1 cells’ various features and average values with standard error of the mean (SEM).

Parameters	Average value ± SEM (n=84)
Circularity	0.2 ± 0.3
Volume	$3054.7\pm 176.7\;\mu m^{3}$
Projected area	$1125.4\pm 71.7\;\mu m^{2}$
Dry mass	17.0 ± 1.0 p gr
Surface area	$29,107.4\pm 2600.1\;\mu m^{2}$
Surface area to volume ratio	9.7±0.6 1/μm
Surface area to dry mass ratio	$1742.8\pm 116.9\;\mu m^{2}/p\;\mathrm{gr}$
Projected area to volume ratio	0.4±0.1 1/μm
Height variance	$4.5\pm 0.4\;\mu m^{2}$
Height kurtosis	2.2 ± 0.1
Height skewness	0.2 ± 0.1
Eccentricity	0.3 ± 0.01
Perimeter	833.5±54.9 μm
Perimeter to area ratio	0.7±0.1 1/μm
Complexity score	0.4 ± 0.02

**Table 4:** Significance test results for the control group in 2 h, including mean difference ± SEM.

Parameters	p-value	Sig.	Mean diff. ± SEM
Circularity	0.715	No	0.0 ± 0.3
Volume	0.872	No	$-6.0\pm 90.7\;\mu m^{3}$
Projected area	0.720	No	$-2.0\pm 29.9\;\mu m^{2}$
Dry mass	0.872	No	−0.03 ± 0.5 p gr
Surface area	0.925	No	$316.4\pm 672.4\;\mu m^{2}$
Surface area to volume ratio	0.562	No	0.5±0.4 1/μm
Surface area to dry mass ratio	0.562	No	$89.1\pm 63.6\;\mu m^{2}/p\;\mathrm{gr}$
Projected area to volume ratio	0.786	No	0.0±0.1 1/μm
Height variance	0.057	No	$0.4\pm 0.4\;\mu m^{2}$
Height kurtosis	0.099	No	0.1 ± 0.1
Height skewness	0.535	No	0.0 ± 0.1
Eccentricity	0.217	No	−0.02 ± 0.01
Perimeter	0.996	No	−10.0±30.0 μm
Perimeter to area ratio	0.239	No	−0.0±0.1 1/μm
Complexity score	0.100	No	−0.02 ± 0.1

### 3.2 Laser-Induced Shockwave Group

In the LIS group, AST1 cells were imaged at multiple time points to capture both immediate and longer-term morphological changes. Imaging began 20 min before LIS to establish baseline conditions, with an additional image taken 1 min before LIS to serve as a final baseline reference. Following the application of the shockwave, a single image was captured 10 min post-LIS to monitor immediate cellular responses. Afterward, cells were imaged at 20-min intervals for the next 2 h to assess longer-term morphological changes. A total of 204 cells from four dishes were analyzed using QPM, allowing for a detailed assessment of dynamic responses to mechanical stress.

#### 3.2.1 Immediate responses post-LIS

As shown in Table 5, significant morphological changes were observed immediately after LIS. Key features such as surface area (p<0.001), perimeter (p<0.001), and surface area to volume ratio (p<0.001) decreased within 10-min post-shockwave, reflecting an acute cellular adaptation to mechanical stress. This reduction in surface area, perimeter, and surface area to volume ratio suggests that the cells may have rapidly retracted their branches, becoming more compact in response to LIS. In contrast, circularity (p<0.001) and volume (p<0.001) increased significantly, indicating that the cells adopted a more spherical morphology, potentially as a stabilizing response to mechanical stress. Other features, such as height variance (p<0.001) and height skewness (p<0.001), showed fluctuations, suggesting changes in cell surface roughness and topographical characteristics.

**Table 5:** Statistical parameters for the cells 1 min before LIS versus 10 min after LIS.

Parameters	p-value	Sig.	Mean diff. ± SEM
Circularity	<0.001	Yes	0.03 ± 0.01
Volume	<0.001	Yes	$352.0\pm 94.5\;\mu m^{3}$
Projected area	<0.001	Yes	$-167.6\pm 25.5\;\mu m^{2}$
Dry mass	<0.001	Yes	2.0 ± 0.5 p gr
Surface area	<0.001	Yes	$-3708.6\pm 752.2\;\mu m^{2}$
Surface area to volume ratio	<0.001	Yes	−2.1±0.4 1/μm
Surface area to dry mass ratio	<0.001	Yes	$-375.5\pm 71.0\;\mu m^{2}/p\;\mathrm{gr}$
Projected area to volume ratio	<0.001	Yes	−0.1±0.01 1/μm
Height variance	<0.001	Yes	$2.2\pm 0.3\;\mu m^{2}$
Height kurtosis	0.307	No	0.0 ± 0.1
Height skewness	0.015	Yes	0.0 ± 0.1
Eccentricity	0.050	No	0.0 ± 0.01
Perimeter	<0.001	Yes	−128.6±22.5 μm
Perimeter to area ratio	0.475	No	0.0±0.1 1/μm
Complexity score	0.384	No	0.0 ± 0.01

#### 3.2.2 Longer-term changes and recovery

Over the 2-h observation period, several features exhibited a trend toward recovery, although not all returned to baseline values. As indicated in Table 6, features such as surface area to volume ratio (p=0.215) and projected area to volume ratio (p=0.333) showed no significant differences compared with their pre-LIS state, suggesting that the cells were gradually reverting to their original morphology. Perimeter also showed an upward trend during recovery, indicating that as the cells may be regaining mechanical stability, they begin to extend their branches again, leading to an increase in cell perimeter (see Table 7).

**Table 6:** Statistical parameters for the cells 1 min before LIS versus 2 h after LIS.

Parameters	p-value	Sig.	Mean diff. ± SEM
Circularity	<0.001	Yes	0.04 ± 0.01
Volume	0.034	Yes	$-46.7\pm 72.8\;\mu m^{3}$
Projected area	0.082	No	$-52.7\pm 27.4\;\mu m^{2}$
Dry mass	0.034	Yes	−0.26 ± 0.4 p gr
Surface area	0.002	Yes	$-1262.8\pm 688.2\;\mu m^{2}$
Surface area to volume ratio	0.215	No	0.8±0.5 1/μm
Surface area to dry mass ratio	0.215	No	$142.4\pm 92.5\;\mu m^{2}/p\;\mathrm{gr}$
Projected area to volume ratio	0.333	No	0.00±0.01 1/μm
Height variance	0.057	No	$0.2\pm 0.2\;\mu m^{2}$
Height kurtosis	0.273	No	0.0 ± 0.1
Height skewness	0.508	No	0.0 ± 0.1
Eccentricity	0.593	No	−0.01 ± 0.01
Perimeter	0.002	Yes	−80.8±22.9 μm
Perimeter to area ratio	0.002	Yes	−0.03±0.01 1/μm
Complexity score	<0.001	Yes	−0.05 ± 0.01

**Table 7:** Statistical parameters for the cells 10 min after LIS versus 2 h after LIS.

Parameters	p-value	Sig.	Mean diff. ± SEM
Circularity	0.419	No	0.01 ± 0.01
Volume	<0.001	Yes	$-398.7\pm 73.4\;\mu m^{3}$
Projected area	<0.001	Yes	$114.8\pm 25.6\;\mu m^{2}$
Dry mass	<0.001	Yes	−2.2 ± 0.4 p gr
Surface area	<0.001	Yes	$2445.8\pm 438.6\;\mu m^{2}$
Surface area to volume ratio	<0.001	Yes	2.9±0.3 1/μm
Surface area to dry mass ratio	<0.001	Yes	$517.8\pm 54.9\;\mu m^{2}/p\;\mathrm{gr}$
Area to volume ratio	<0.001	Yes	0.09±0.01 1/μm
Height variance	<0.001	Yes	$-2.0\pm 0.3\;\mu m^{2}$
Height kurtosis	0.147	No	0.0 ± 0.1
Height skewness	0.097	No	0.0 ± 0.1
Eccentricity	0.036	Yes	−0.02 ± 0.01
Perimeter	0.075	No	47.8±21.6 μm
Perimeter to area ratio	<0.001	Yes	−0.04±0.01 1/μm
Complexity score	0.001	Yes	−0.03 ± 0.01

**Fig. 6:**
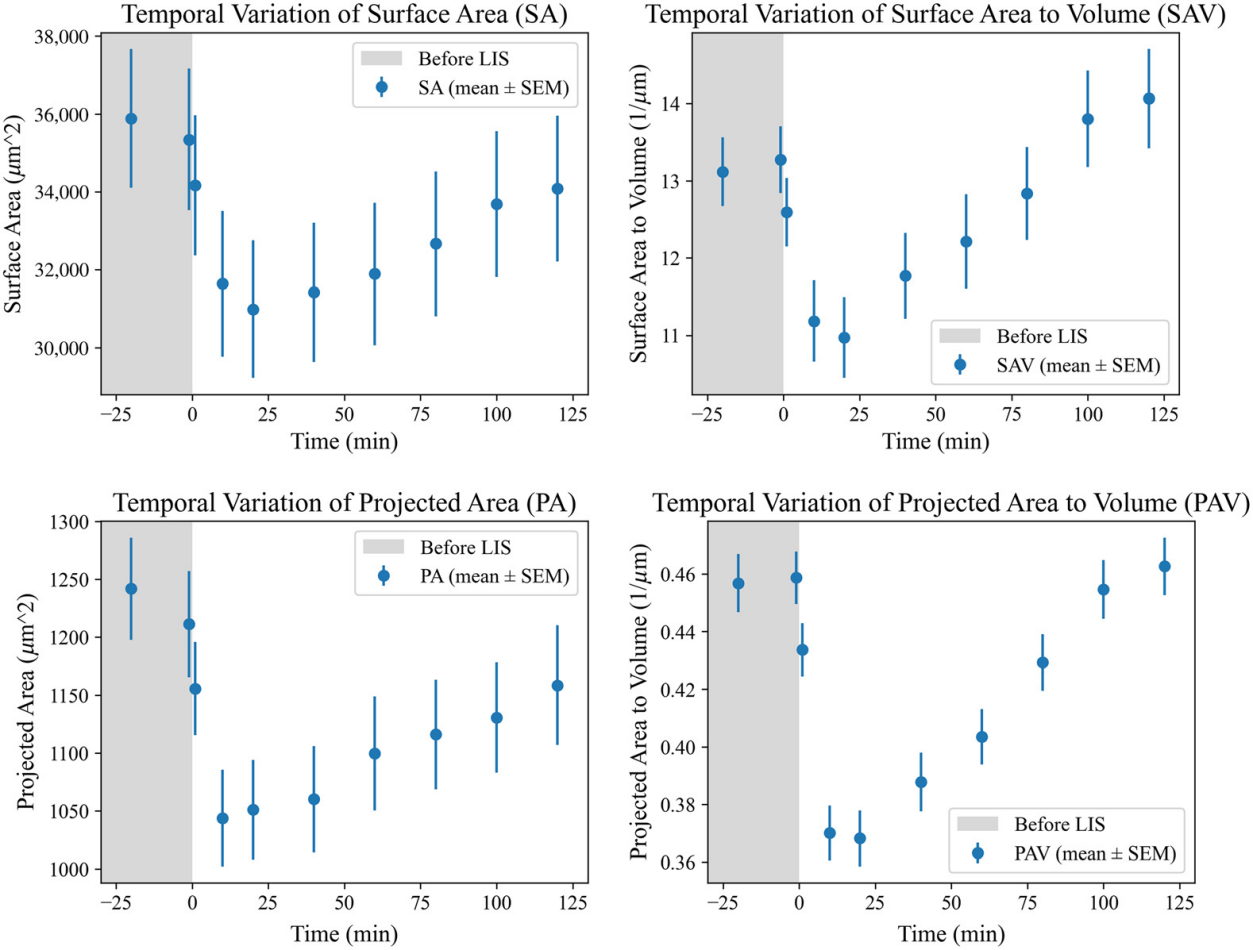
Temporal analysis of key morphological and geometric features, including surface area, surface area to volume ratio, projected area, and projected area to volume ratio, measured at different time points with respect to exposure to a laser-induced shockwave. AST1 cells exhibited linear changes in these features over time, demonstrating an initial response to the shockwave, followed by a gradual return to values close to their pre-shockwave state.

Although surface area and volume had not yet returned to their baseline levels, both exhibited clear trends toward recovery, as confirmed by the Mann–Kendall test, which identified significant time-dependent trends in volume ($\tau_{b}=-1$, p<0.005), surface area ($\tau_{b}=1$, p<0.005), and surface area to volume ratio ($\tau_{b}=1$, p<0.005). The temporal variation of key morphological features—surface area, surface area to volume ratio, projected area, and projected area to volume ratio—reveals a more nuanced recovery trajectory over time. As shown in Fig. 6, all mentioned features initially decreased after the shockwave, followed by a gradual upward trend over the 2-h period. These ratios initially decreased, suggesting cellular compaction, but began to steadily increase from around 40 min post-LIS, indicating a gradual return to pre-shockwave morphology. We can observe a gradual decrease in the scope of the changes in 100 to 120 min after the exposure to shockwave, which may suggest that the cells are reaching morphological stability.

## 4 Discussion

This study aims to explore the morphological changes that occur in astrocyte cells in response to laser-induced shockwaves, providing valuable insights into the dynamics of cellular responses to mechanical stimuli. The motivation behind this research lies in understanding the microscopic consequences of traumatic events, particularly TBI, at the cellular level. Focusing on astrocytes, the most abundant cells in the central nervous system, we simulate TBI conditions *in vitro* by subjecting cells to controlled shear stress through laser-induced shockwaves. For accurate measurement of morphological alterations, we utilized QPM, which is capable of measuring samples with axial resolution down to 1 nm.^28^ This enables precise, quantitative measurement of features such as surface area, volume, and dry mass, metrics which are difficult or impossible to obtain with conventional “2D” imaging techniques. QPM is particularly valuable in this study as it allows us to measure the minute structural responses of astrocytes to mechanical stress without the need for invasive labeling techniques. This setup, integrated with time-lapse imaging, enhances the resolution and quantitative capabilities of the experiment, making it ideal for studying morphological changes following mechanical insult.

The experimental setup, involving a control group and an LIS group, ensures a systematic exploration of the effects of shockwaves on astrocyte morphology. In the control group, environmental conditions such as humidity and ${\mathrm{CO}}_{2}$ levels were carefully regulated to minimize external factors that could influence cell behavior. Comprehensive characteristics of AST1 cells were measured using QPM, which provided a robust baseline for comparison with the shockwave group. Over the 2-h imaging period, astrocyte morphology remained stable in the control group, confirming that any changes observed in the LIS group were due to mechanical stress rather than environmental factors.

In the LIS group, the investigation of AST1 cells uncovered immediate and sustained morphological changes, providing insights into dynamic cellular adaptations. Following LIS, the AST1 cells rapidly transitioned to a more circular shape, accompanied by significant changes in volume, surface characteristics, and height features. These changes could not be easily quantified using conventional imaging techniques such as phase-contrast or widefield microscopy. This trend in surface-based features aligns with cytoskeletal remodeling processes described in earlier studies,^29^^,^^30^ where cells subjected to mechanical stress undergo rapid initial compaction followed by cytoskeletal reorganization over time. The initial reduction in surface area and perimeter after LIS may reflect a protective cellular response to mechanical stress, as the cells adopt a more compact morphology. Over time, features such as projected area and surface area, as well as their ratios to volume, showed upward trends, indicating that the cells gradually extended their branches and regained mechanical stability. These changes suggest the cells were progressively returning to their original structure as the cytoskeleton reorganized, consistent with findings from brain cell recovery studies, where growth cones extend toward sites of injury to facilitate recovery.^29^^,^^30^

The observed changes in geometric features suggest small-scale cytoskelelatal reorganization is taking place directly after damage which aligns with previous work in this area.^29^ However, further investigations into mechanotransduction pathways are crucial to understanding the processes driving these adaptations. Importantly, such feature changes are distinct from the “balling up” behavior which can be observed by the eye as dying cells. The ability to extract features such as cell area, dry mass, and volume using QPM offers reproducible metrics for understanding previously unobservable cellular characteristics, opening the door to broader applications in TBI research and neurodegenerative disorders. For example, in diseases such as Huntington’s, where neurons exhibit protein aggregation, these methods can quantify changes in cell morphology related to pathology.^31^

However, challenges remain in astrocyte segmentation due to their complex branching structures and tendency to form clusters. Automated tools, such as the watershed algorithm, can assist in segmentation but often fall short in detecting fine astrocytic processes. Deep learning and machine learning techniques show potential in overcoming these challenges by learning complex data patterns and reducing manual intervention. However, they require extensive training data and computational resources, and interpretability can be an issue, particularly in critical applications where decision transparency is necessary.^32^^,^^33^ Future work will extend this study by employing longer observation periods to capture the complete astrocyte recovery timeline, better enhancing our understanding of their morphological responses and mechanical stability following shockwave exposure. In addition, investigations will be conducted on mixed cultures of astrocytes and neurons to better understand the complex interactions among cell types under mechanical stress. These advancements will further illuminate the underlying mechanisms of cellular response to injury and provide new avenues for therapeutic interventions in neurotrauma and related conditions.

## 5 Conclusion

This study provides significant insights into the dynamic morphological responses of astrocytes to laser-induced shockwaves, simulating traumatic brain injury conditions *in vitro*. Using QPM, we were able to measure crucial features such as surface area, volume, and height variance, offering a level of precision and quantitative detail that is not achievable with traditional fluorescence microscopy. Our findings demonstrate an immediate rounding of the cell structure, followed by a gradual recovery marked by branch regrowth and surface stabilization. Previous studies have shown the early stages of actin-based cytoskeletal remodeling at the site of structural tubulin damage, suggesting a similar process to how astrocytes may be recovering under mechanical stress.^29^

The use of QPM in this study proved to be a powerful tool for non-invasive, high-precision measurement of cellular responses, making it applicable for broader investigations into mechanotransduction, TBI, and neurodegenerative diseases. Future work should focus on extended time frames and more complex cellular environments to further elucidate the role of astrocytes in mechanical injury and recovery.

## Data Availability

The data that support the findings of this study are available from the corresponding author upon reasonable request.
